# The Effect of Anodizing Bath Composition on the Electronic Properties of Anodic Ta-Nb Mixed Oxides

**DOI:** 10.3390/nano12244439

**Published:** 2022-12-14

**Authors:** Giada Tranchida, Andrea Zaffora, Francesco Di Franco, Monica Santamaria

**Affiliations:** Department of Engineering, University of Palermo, Viale delle Scienze, 90128 Palermo, Italy

**Keywords:** anodizing, capacitance, electrolytic capacitors, mixed oxides, Ta oxide, Nb oxide

## Abstract

Anodic oxides were grown to 50 V on Ta-Nb sputtering deposited alloys, with high Nb content, in acetate ions containing an aqueous solution to study the effect of the anodizing bath composition on anodic layers’ dielectric properties. Photoelectrochemical measurements proved the presence of a photocurrent in the band gap of photon energy lower than oxides, due to optical transitions involving localized electronic states as a consequence of acetate ions incorporation. Flat band potential value estimates assessed the insulating nature of the anodic oxides grown in the acetate buffer solution. Differential capacitance measurements showed that the highest capacitance value was measured for the sample grown on Ta-66%Nb. This capacitance value was higher with respect to those estimated for pure Ta and pure Nb anodic layers and with respect to the same alloy anodized in NaOH solution, i.e., acetate-free anodizing bath.

## 1. Introduction

Anodizing is a low-temperature, low-cost electrochemical process which allows the growth of oxide layers of tunable composition and properties on the surface of the so-called valve metals (e.g., Nb, Ta, Al, etc.) and valve metal alloys. By a careful choice of process parameters, it is possible to fabricate oxides with very well-controlled features. In fact, controlling anodizing conditions has a direct influence on oxide layer thickness and on its crystalline or amorphous nature, while the composition of an electrolytic bath and the base alloy affect the composition of the oxides. Moreover, it is important to consider the effect related to the possible incorporation of foreign species coming from the solution into the anodic layer and the role of pH and aggressive ions in determining the morphology of the growing layer, changing from barrier to porous films [1,2,3]. Since anodizing is a well-established industrial process, understanding how the operating parameters affect the properties of the anodic oxides, therefore, their performance as dielectric materials is a key point.

Anodizing is currently used as a formation step in the manufacturing process of electrolytic capacitors to grow an oxide layer on the surface of aluminum or tantalum, since Al_2_O_3_ and Ta_2_O_5_ are the main dielectric materials used in electrolytic capacitors. The operating conditions of the anodizing process, e.g., formation voltage, temperature, anodizing bath composition, must be carefully selected since they can affect morphological and electronic properties of the anodic oxides and, consequently, can affect the performance of the final device in terms of specific capacitance and leakage current. Therefore, at the industrial level, high dielectric constant and insulating materials were chosen for high-performance electrolytic capacitors. Ta_2_O_5_ has a high dielectric constant (ε = 22−30 [4,5]) and a band gap of 3.9–4.3 eV [6,7] while Al_2_O_3_ is a very insulating material (band gap between 6.2 and 8 eV [8,9]) but with a low dielectric constant (ε = 9 [10]).

In the last decades, research efforts focused on the study of oxides with high capacitance and insulating nature to meet both material’s requirements for high-performance electrolytic capacitors. It was proved that a valuable strategy to increase the dielectric constant value of Ta_2_O_5_ is anodizing Ta alloys, as Ta-Nb alloys, for example, since pure Nb_2_O_5_ has higher dielectric constant (ε ≈ 50 [11,12,13]) with respect to pure Ta oxide, obtaining mixed Ta-Nb oxides with a higher dielectric constant and capacitance than those of Ta_2_O_5_ [12,13,14]. Mixed oxides can also be used as functional materials for many other applications, such as magnetic/electronic devices [15,16], for corrosion protection [17] or as energy storage materials [18].

Anodizing sputtering deposited Ta-Nb alloys allows for the growth of mixed oxides by a cooperative transport of O^2−^ ions from the oxide/electrolyte interface toward the metal/oxide interface and cations in the opposite direction. According to the transmission electron microscopy images of ultramicrotome cross sections of anodized alloys, these oxides are amorphous, featureless, compact and uniform, as expected for barrier layers on valve metals such as tantalum and niobium [3,14]. According to Rutherford Back Scattering analysis, the Ta-Nb ratio in the mixed oxide is almost coincident with that of the base alloys [14].

Moreover, previous studies have established that the incorporation of certain foreign species from the electrolyte during the anodizing process can further increase the dielectric constant of valve metal oxides [19,20,21,22]. However, the incorporation of such species can affect the electronic properties of the anodic oxides changing the density of states (DoS) distribution inside the oxides because it can cause the formation of allowed localized states inside the band gap of the materials. Since the electronic properties of the oxides are directly related to the leakage current, a study on the effect on the electronic properties of the incorporation of foreign ions during anodizing is needed.

In this work, we want to study the effect of acetate incorporation on the electronic properties of 50 V anodic films grown on Ta-Nb sputtering deposited alloys of several compositions by the synergistic use of photoelectrochemical and impedance measurements with a specific interest in the dielectric constant. A survey of the published literature shows that there are previous papers [12,13,14,21,23] focused on anodic films on Ta-Nb alloys but with different thicknesses, compositions and, thus, electronic properties. In this aim, the alloys were anodized in an acetate buffer aqueous solution at a formation voltage of 50 V. Photoelectrochemical measurements were carried out to get information about the electronic properties of the mixed oxides, such as optical band gap, flat band potential and semiconducting/insulating nature. The effect of such incorporation on the dielectric properties was studied by differential capacitance measurements and electrochemical impedance spectroscopy. Finally, leakage current measurements were carried out as a function of anodizing bath composition.

## 2. Materials and Methods

Ta-Nb alloys were deposited by dc magnetron sputtering on glass substrates. Deposition targets were a 99.9% Nb disk (100 mm diameter) and different 99.9% Ta disks (20 mm diameter) located symmetrically on the erosion zone of the Nb target. The sputtering chamber was initially evacuated to ~5 × 10^−5^ Pa and then, the sputtering deposition was carried out in 99.999% argon (~0.3 Pa) at 0.5 A for 600 s. The anodizing process was carried out potentiodynamically at 100 mV s^−1^ up to a formation voltage of 50 V in a 0.1 M Acetic Acid and 0.1 M Sodium Acetate aqueous solution (Acetate Buffer Solution, ABS) at pH = 6. The temperature was set at 25 °C and no mixing action was used.

The experimental set-up employed for the photoelectrochemical investigations consisted of a 450 W UV–VIS xenon lamp coupled with a monochromator (Kratos), which allows monochromatic irradiation of the sample surface through a quartz window of the electrochemical cell. A two-phase lock-in amplifier (EG&G) was used and connected with a mechanical chopper (working at 13 Hz) in order to separate the photocurrent from the total current circulating in the cell due to the potentiostatic control. Photocurrent spectra reported in the text are corrected for the relative photon flux of the light source at each wavelength, so that the photocurrent yield (Q_ph_) is represented in the y-axis in arbitrary current units. All the experiments were performed in air at room temperature. The characterization electrolyte was a 0.1 M Ammonium Biborate (ABE, pH ≈ 8.5) aqueous solution.

For all the electrochemical and photo-electrochemical experiments, the reference electrode was a saturated silver/silver chloride electrode (0 V vs. Ag/AgCl = 0.197 V vs. SHE).

Differential capacitance measurements and Electrochemical Impedance Spectra were recorded in a 0.25 M Na_2_HPO_4_ solution (pH~9) by using a Parstat 2263 (PAR), connected to a PC for the data acquisition. For all the experiments, the counter electrode was a Pt net having a very high surface area. The amplitude of ac voltage signal was 10 mV and the frequency range for EIS measurements was 100 kHz–100 mHz.

## 3. Results and Discussion

Table 1 reports the current density values measured during the potentiodynamic anodizing of Nb, Ta and Nb-Ta alloys at 100 mV s^−1^ in ABS. The current density value increases by increasing the Nb content in the metallic substrate. According to Faraday’s law and in agreement with the high field mechanism for the anodic film’s growth, it is possible to estimate the electric field strength, *E*, from *dV*/*dt*, according to Equation (1):

(1) $$\frac{dV}{dt}=\eta \frac{iEM}{zF\rho }$$

in which *i* is the measured current density during the oxide growth, *M* is the molecular weight of the anodic oxide, *z* is the number of electrons related to the formation of one mole of oxide (i.e., 10), *F* is the Faraday constant, *ρ* is the anodic oxide density and *η* is the oxide growth efficiency. *η* is defined as [12]:

(2) $$\eta =\frac{i_{form}}{i_{tot}}=\frac{i_{form}}{i_{form}+i_{diss}+i_{el}}$$

where *i_form_* is the ionic current density that sustains the oxide film growth, *i_diss_* is the current density due to oxide dissolution, and *i_el_* is the electronic current density. *i_diss_* is expected to be negligible since Ta and Nb oxides are thermodynamically stable at pH = 6, as shown in Pourbaix diagrams relating to Ta and Nb [24], and *i_el_* is negligible due to the blocking character of the oxides [12]. Equation (1) allows us to estimate the electric field strength, E. The reciprocal of the latter is the anodizing ratio, k, that was estimated for the investigated anodic oxides, considering that density and molecular weight values were obtained by averaging *ρ* and *M* values of Nb_2_O_5_ and Ta_2_O_5_ according to the specific alloy composition. Notably, as reported in literature [13,14] and in agreement with the comparable migration rate (transport number) of Nb^5+^ and Ta^5+^ ions during the anodizing process [2], the ratio of Ta/Nb in the anodic oxide is almost coincident with the corresponding ratio in the metal alloy. The knowledge of k is important, since the oxide thickness can be estimated by multiplying the anodizing ratio for the final formation voltage (see Table 1). It is worth mentioning that the anodizing ratio of Nb is higher than that of Ta.

**Figure 1:**
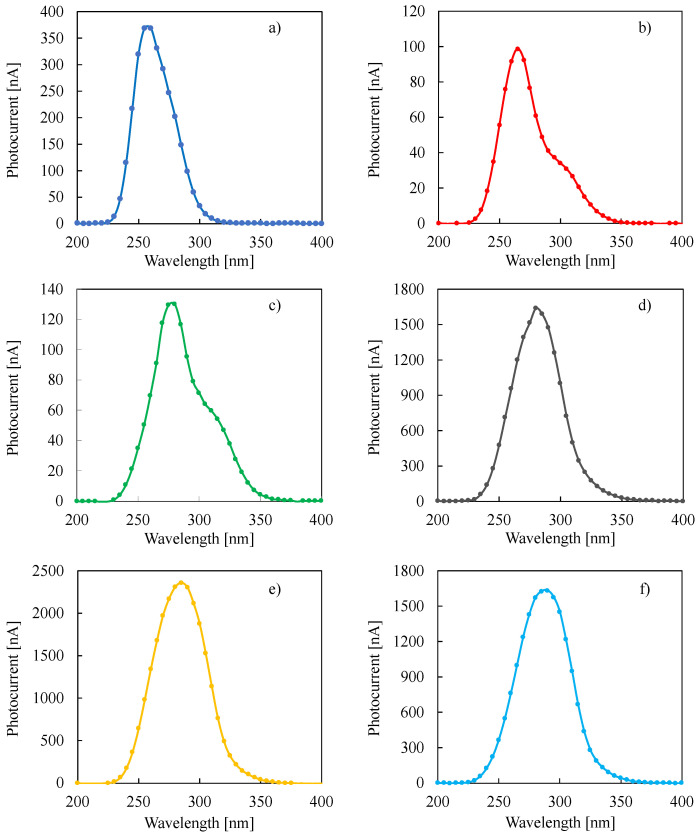
Photocurrent spectra relating to anodic films grown up to 50 V vs. cathode in ABS on (**a**) Ta, (**b**) Ta-19at% Nb, (**c**) Ta-39at% Nb, (**d**) Ta-66at% Nb, (**e**) Ta-85at% Nb and (**f**) Nb, recorded at 8 V vs. Ag/AgCl in 0.1 M ABE.

In order to estimate the optical band gap of anodic oxides, photocurrent spectra (photocurrent vs. irradiating wavelength curves) were recorded at 8 V vs. Ag/AgCl for all the investigated films (see Figure 1). From the photocurrent spectra, the optical band gap value, E_g,opt_, can be estimated according to the following relationship [25]:

(Q_ph_ · hν)^n^ ∝ (hν − E_g,opt_) (3)

valid for photon energy, hν, in the vicinity of the band gap and where Q_ph_ is the photocurrent yield, i.e., photocurrent value corrected for the relative photon flux at each irradiating wavelength.

**Figure 2:**
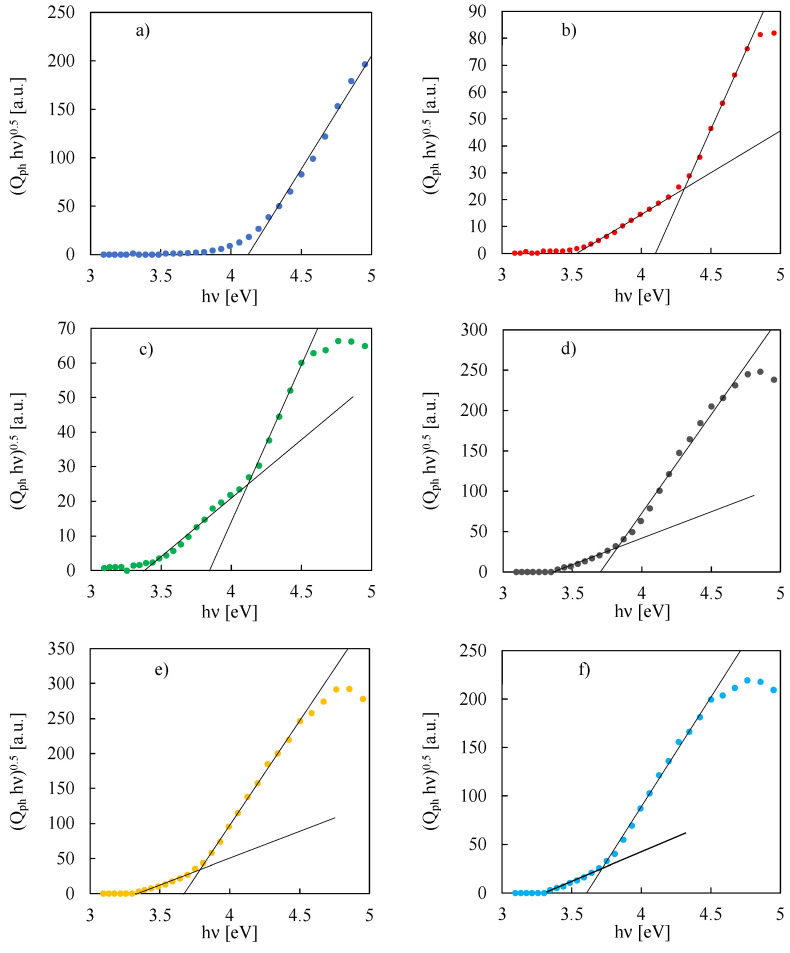
(Q_ph_ · hν)^0.5^ vs. hν plots relating to photocurrent spectra, shown in Figure 1, for anodic films grown up to 50 V vs. cathode in ABS on (**a**) Ta, (**b**) Ta-19at% Nb, (**c**) Ta-39at% Nb, (**d**) Ta-66at% Nb, (**e**) Ta-85at% Nb and (**f**) Nb.

Assuming non-direct optical transitions (n = 0.5), E_g,opt_ can be estimated by extrapolating the so-called Tauc plot, i.e., (Q_ph_ · hν)^0.5^ vs. hν plot, as shown in Figure 2 for the anodic oxides grown on Nb-Ta alloys in ABS.

E_g,opt_ values, estimated according to Equation (3), are reported in Table 2.

For anodic oxide grown on pure Ta, a E_g,opt_ of 4.12 eV was estimated, higher than the typical band gap value of crystalline Ta_2_O_5_, i.e., 4 eV [7,26]. This condition is usually associated with the formation of an amorphous film and, more specifically, with the lack of long-range order causing a distribution of localized states close to the conduction band and to the valence band [27]. It is worth noting that, for the other anodic oxides except for Ta_2_O_5_, it was possible to estimate two different absorption threshold values. More specifically, the higher ones are related to the energy transitions between the valence and conduction band since they are close to those estimated for Ta-Nb mixed oxides grown in NaOH aqueous solution, i.e., in absence of incorporation phenomena from the electrolyte [12]. Conversely, the lower absorption threshold values are due to energy transitions another then band-to-band transitions. In fact, it was demonstrated that the incorporation of foreign species, namely anions, to the electrolytic bath during the anodizing process can lead to the formation of localized states inside the band gap of the semiconducting material, with consequent photocurrent generation also for irradiating photons with energy lower than E_g,opt_ [21,22,28,29,30]. In the case of Ta-Nb anodic oxides grown to 50 V in acetate-containing solution, it can be inferred that the lower absorption threshold, E_LS_, is due to energy transitions between localized states (due to acetate ions incorporation) and extended states. Whether the anodic oxide is used as a dielectric in electrolytic capacitors, understanding the energy location inside the gap of these localized states is a key aspect, since they can induce the formation of percolation paths for the electronic conduction with the detrimental effect of increasing the leakage current. The lower absorption threshold values range between 3.31 eV for the anodic oxide grown on Nb and 3.54 eV for the oxide grown on Ta-19at.%Nb (see Table 2). In agreement with this result, it has been recently demonstrated that organic ion incorporation can happen during anodizing of valve metals [19,31,32,33] with the generation of localized states inside the band gap of the oxide [22].

**Figure 3:**
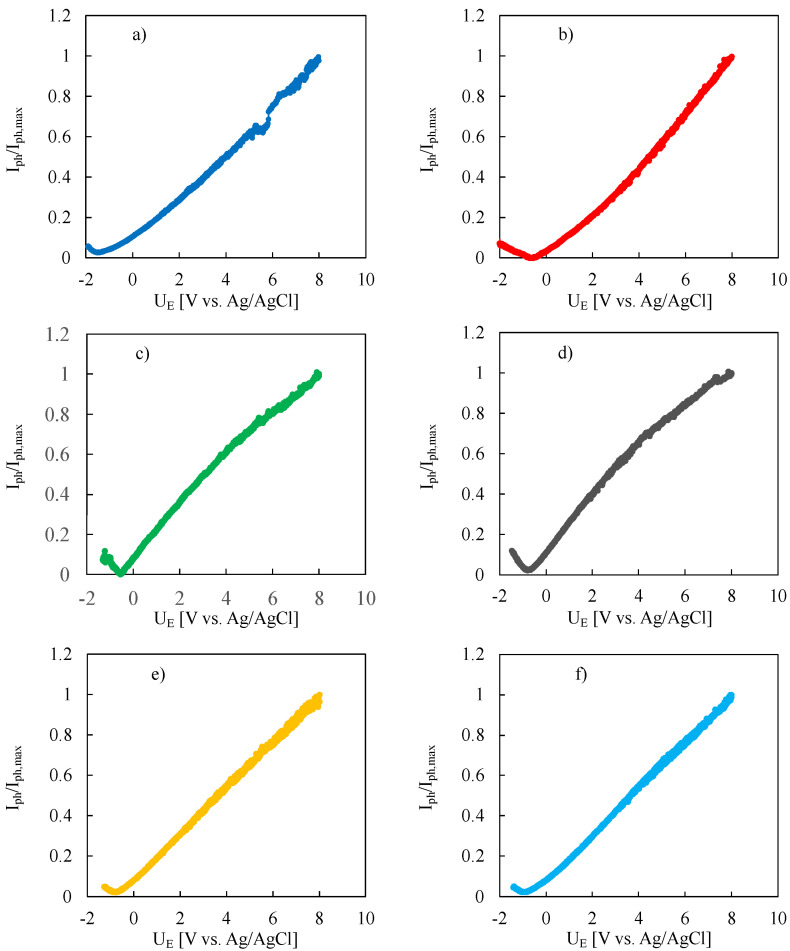
Normalized photocharacteristics relating to anodic films grown up to 50 V in ABS on (**a**) Ta, (**b**) Ta-19at% Nb, (**c**) Ta-39at% Nb, (**d**) Ta-66at% Nb, (**e**) Ta-85at% Nb and (**f**) Nb. Irradiating wavelength: 260 nm. Potential scan rate: 10 mV s^−1^. Solution: 0.1 M ABE.

I_ph_ vs. U_E_ curves at constant irradiating photon wavelength (photocharacteristics) were recorded in order to study the dependence of photocurrent on the applied potential. Normalized photocharacteristics (I_ph_/I_ph,max_) at 260 nm (i.e., 4.76 eV) for all investigated oxides are shown in Figure 3.

**Figure 4:**
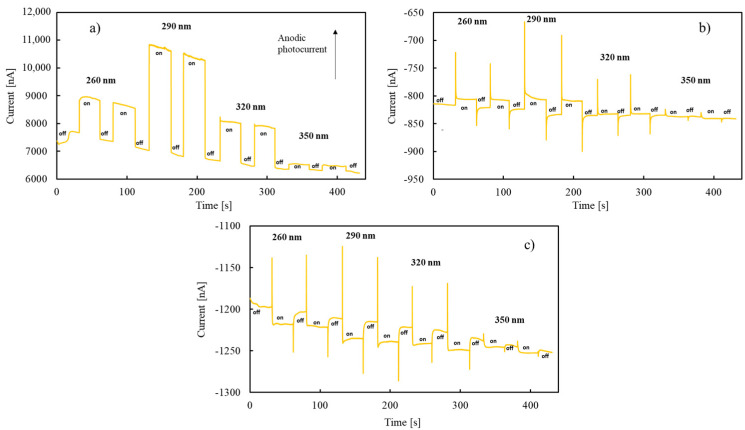
Total current circulating without irradiation (off) and with irradiation (on) in the oxide grown on Ta-85at%Nb to 50 V at λ = 260, 290, 320 and 350 nm by polarizing the electrode at (**a**) 8 V, (**b**) −0.4 V and (**c**) −0.5 V vs. Ag/AgCl.

By sweeping the electrode potential from 8 V vs. Ag/AgCl in the cathodic direction, the measured anodic photocurrent decreased because of decreasing of the applied electric field across the anodic layer. Moreover, the photocurrent sign changed from anodic to cathodic (photocurrent phase angle not shown) for sufficient cathodic electrode potentials. This behaviour is typical of insulating materials, i.e., materials with Fermi level far enough from conduction/valence band edges, for which it is possible to generate both anodic and cathodic photocurrent depending on the direction of the applied electric field across the oxide film. The latter is directly related to the applied electrode potential and to its difference with respect to the flat band potential, U_FB_, of the material. Photocurrent sign inversion potential, U_inv_, detected during photocharacteristics recording is expected to be close to the U_FB_ value. However, a better and more reliable way to estimate the U_FB_ value can be done by recording current transients with and without sample irradiation at different electrode potentials, as shown in Figure 4 where current vs. time curves are reported for the anodic oxide grown on Ta-85at%Nb to 50 V at three different electrode potentials, i.e., 8 V, −0.4 V and −0.5 V vs. Ag/AgCl, at λ = 260, 290, 320 and 350 nm.

At 8 V vs. Ag/AgCl (high anodic polarization), the current suddenly increased soon after sample irradiation, generating an anodic photocurrent (see Figure 4a). At −0.4 V vs. Ag/AgCl, the photocurrent was still anodic but the stationary value was measured after a spike of the current after irradiation (see Figure 4b), suggesting the occurrence of strong recombination phenomena of the photogenerated carriers [29]. At −0.5 V vs. Ag/AgCl, the measured current had an anodic spike; then, it reached a stationary value with a cathodic photocurrent (see Figure 4c), meaning that −0.5 V vs. Ag/AgCl was an electrode potential more cathodic with respect to the flat band potential and that U_FB_ is more anodic than U_inv_. The estimated U_FB_ values, according to current transients, for all the anodic oxides are reported in Table 3.

The difference between U_FB_ and U_inv_ can be explained by considering that localized states inside the band gap of the oxides can also behave as traps for the photogenerated carriers, modifying the distribution of the electric field at electrode/electrolyte interface [5]. For 50 V anodic oxides grown in ABS, the U_FB_ values resulted to be more positive (anodic) than those estimated for 50 V films grown on the same alloys in the alkaline solution (i.e., 0.1 M NaOH) [12]. This result suggests that anodic oxides grown in ABS solution are more insulating with respect to the same oxides grown in a NaOH solution.

**Figure 5:**
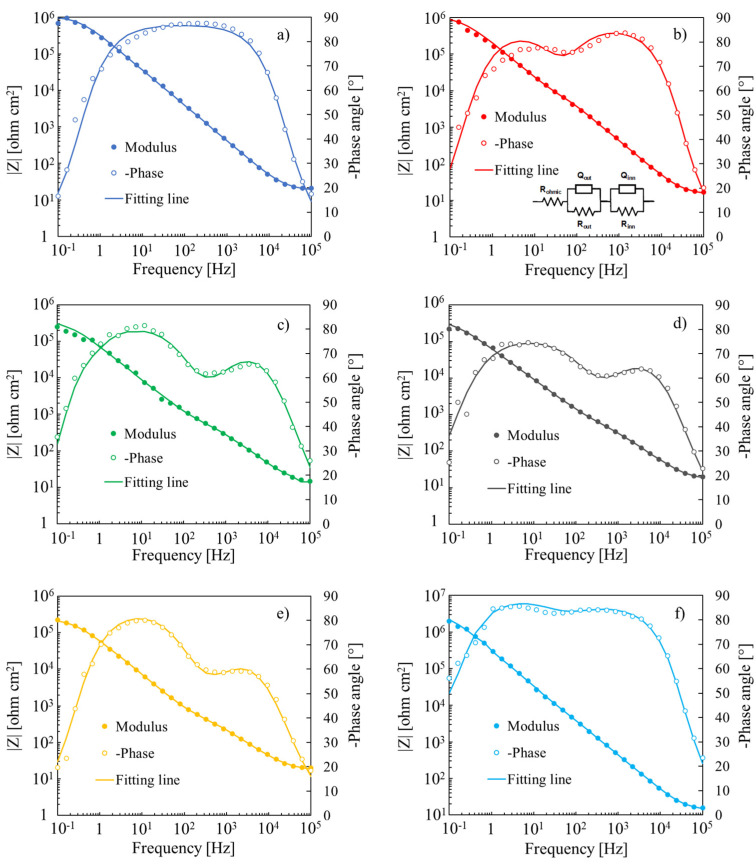
Bode representation of EIS spectra relating to all the investigated 50 V anodic oxides grown on (**a**) Ta, (**b**) Ta-19at% Nb, (**c**) Ta-39at% Nb, (**d**) Ta-66at% Nb, (**e**) Ta-85at% Nb and (**f**) Nb, recorded at U_E_ = 8 V vs. Ag/AgCl in 0.25 M Na_2_HPO_4_. Inset: EEC employed to model the metal/anodic oxide/electrolyte interfaces.

In Figure 5a–f, the electrochemical impedance spectra (EIS) relating to all the investigated 50 V anodic oxides recorded at 8 V vs. Ag/AgCl are shown. In order to fit the experimental impedance spectra by properly modeling the electrochemical behaviour of the metal/anodic layer/electrolyte systems, the equivalent electrical circuit (EEC) shown in the inset of Figure 5b was used.

For all of the anodic oxides except for Ta_2_O_5_, a two-time constant EEC was used, i.e., the electrolyte resistance R_el_ in series with two parallel (RQ), where Q was a constant phase element (CPE) introduced to model a non-ideal capacitance behaviour. This EEC is related to a double-layered structure of the anodic oxides grown in ABS solution, taking into account the possibility that the incorporation of acetate ions from the electrolyte during the anodizing process is limited to just an outer layer, as happens with other foreign species incorporation from the electrolyte [28,31,34,35,36,37]. The fitting parameters of EIS spectra are reported in Table 4.

**Figure 6:**
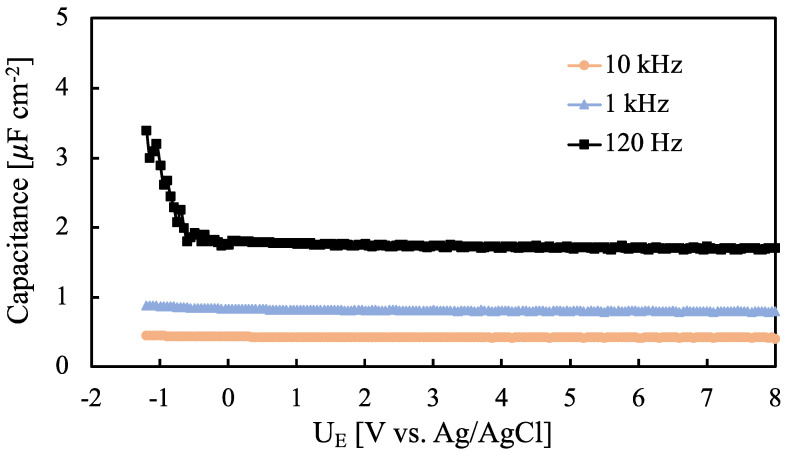
Measured series capacitance relating to anodic oxide grown up to 50 V on Ta-66at.%Nb recorded at 10 kHz, 1 kHz and 120 Hz in 0.25 M Na_2_HPO_4_ solution (pH~9).

In order to get information on the dielectric properties of investigated anodic oxides, differential capacitance measurements were carried out at different ac frequencies. In Figure 6 we report measured series capacitance, C_M_, curves relating to 50 V oxide on Ta-66at.%Nb recorded at 10 kHz, 1 kHz and 120 Hz in 0.25 M Na_2_HPO_4_ solution (pH~9).

**Figure 7:**
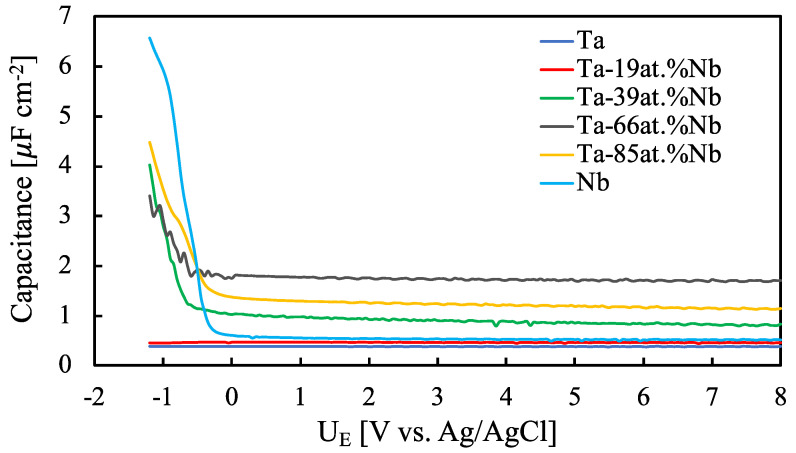
Measured series capacitance relating to all the investigated anodic oxides grown up to 50 V at 120 Hz in 0.25 M Na_2_HPO_4_ solution (pH~9).

It is noteworthy to mention that C_M_ depends on the ac frequency, as typical of amorphous materials. The dependence of C_M_ on the electrode potential is almost negligible as typical for insulating materials unless the anodic oxide is tested under a strong cathodic polarization. C_M_ measured at 120 Hz for all the investigated anodic oxides is shown in Figure 7.

Estimating the capacitance of the anodic layer at 120 Hz is particularly important since usually the commercial capacitor’s performance indicators (e.g., capacitance, ripple current) are evaluated at this frequency. It is interesting to note that anodic oxides became more polarizable approaching flat band potential value by increasing Nb content in the film. The highest measured capacitance, evaluated under high-band-bending conditions (i.e., at 8 V vs. Ag/AgCl), was measured for the anodic oxide grown on Ta-66at%Nb alloy, 1.7 µF cm^−2^. This value is more than four times higher than that measured for the anodic oxide on Ta and more than three times higher than that measured for the anodic oxide on Nb. It is worth mentioning that this capacitance value is also about three times higher than that of the anodic oxide grown in NaOH solution [21].

**Figure 8:**
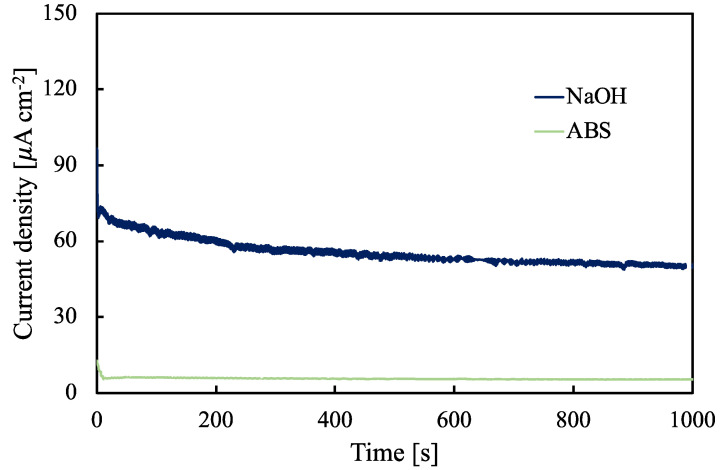
Leakage current measurement for the anodic oxides grown on Ta-66at%Nb alloy in NaOH and ABS solutions at 50 V by applying 37.5 V for 1000 s.

An important parameter for the evaluation of a capacitor performance is the leakage current, which we measured for the anodic oxides grown on Ta-66at%Nb in NaOH and ABS solutions by applying 37.5 V, i.e., 75% of formation voltage. Figure 8 shows the current density vs. time curves related to the leakage current measurement.

The leakage current measured for the anodic oxide grown in NaOH solution, i.e., non-incorporating anodizing bath, was 50 mA cm^−2^ whilst that measured for the anodic oxide grown in ABS was 5 mA cm^−2^, i.e., 10 times lower. This result is in agreement with the higher insulating nature of the anodic oxide grown in ABS, showing a better blocking behaviour that is a crucial feature for the usage of this oxide as the dielectric in electrolytic capacitors.

## 4. Conclusions

Anodic oxides were grown potentiodynamically on Ta-Nb alloys with several compositions to 50 V in acetate-containing aqueous electrolyte with the goal to obtain dielectric materials with high specific capacitance to be used in electrolytic capacitors.

Photoelectrochemical measurements assessed a redshift in the optical absorption for all the anodic oxides, except for anodic oxide grown on pure Ta, with a second optical threshold value ranging between 3.31 and 3.54 eV. This redshift can be explained by taking into account energy transitions another then band-to-band transitions. In particular, acetate ions incorporated during the anodizing process can lead to the generation of localized electronic states inside the band gap of the anodic oxides, that are responsible for the second detected optical absorption threshold value. Flat band potential value estimates assessed a higher insulating nature of the anodic oxides grown in acetate-containing electrolytes with respect to those grown in the NaOH solution, i.e., non-incorporating anodizing bath.

Electrochemical Impedance Spectra showed a two-time constant behavior that confirmed the presence of a layered structure due to the incorporation of foreign species, namely acetate ions, from the electrolyte during the anodizing process. Differential capacitance measurements assessed the highest capacitance value for the anodic oxide grown on Ta-66at%Nb alloy, i.e., 1.7 µF cm^−^^2^, the capacitance value that is higher than those measured for pure Ta and Nb oxide, 0.4 µF cm^−^^2^ and 0.5 µF cm^−^^2^, respectively, and higher than that measured for the same oxide grown in a NaOH solution. These conclusions suggest that anodizing in acetate-containing electrolytes can be a valuable strategy to fabricate high-performance dielectric materials for electrolytic capacitors.

## Figures and Tables

**Table 1:** Anodic oxides growth parameters, including estimated oxides thickness.

Sample	i [µA cm^−2^]	E [MV cm^−1^]	k [nm V^−1^]	d [nm]
Nb	410	3.7	2.7	135
Ta-85at.%Nb	395	4.0	2.5	124
Ta-66at.%Nb	370	4.5	1.9	112
Ta-39at.%Nb	363	4.7	2.1	106
Ta-19at.%Nb	304	5.8	1.7	86
Ta	289	6.2	1.6	81

**Table 2:** Band gap and second optical absorption threshold values estimated according to Equation (3) relating to all the anodic oxides.

Base Alloy	E_g,opt_ [eV]	E_LS_ [eV]
Ta	4.12	n.d.
Ta-19at.%Nb	4.10	3.54
Ta-39at.%Nb	3.85	3.38
Ta-66at.%Nb	3.70	3.37
Ta-85at.%Nb	3.67	3.34
Nb	3.61	3.31

**Table 3:** Flat band potential values estimated with current transients recorded in 0.1 M ABE under constant irradiating wavelength relating to all the anodic oxides.

Base Alloy	U_FB_ vs. (Ag/AgCl) [V]
Ta	−0.90
Ta-19at.%Nb	−0.45
Ta-39at.%Nb	−0.40
Ta-66at.%Nb	−0.45
Ta-85at.%Nb	−0.45
Nb	−0.50

**Table 4:** Fitting parameters relating to EIS spectra of all the investigated 50 V anodic films using EEC shown in Figure 5b. In the case of Ta anodic oxide, R_inn_ is R_ox_.

Base Alloy	R_ohmic_ [Ω cm^2^]	R_out_ [Ω cm^2^]	Q_out_ [S s^α^ cm^−2^]	α	R_inn_ [Ω cm^2^]	Q_inn_ [S s^α^ cm^−2^]	α
Ta	19	-	-	-	1 × 10^6^	5.0 × 10^−7^	0.97
Ta-19at.%Nb	15	1 × 10^6^	8.3 × 10^−7^	0.95	2 × 10^3^	1.3 × 10^−6^	0.99
Ta-39at.%Nb	12	4 × 10^5^	2.5 × 10^−6^	0.92	3 × 10^2^	3.1 × 10^−6^	0.85
Ta-66at.%Nb	16	4 × 10^5^	3.1 × 10^−6^	0.86	3 × 10^2^	3.7 × 10^−6^	0.84
Ta-85at.%Nb	17	2 × 10^5^	2.8 × 10^−6^	0.94	3 × 10^2^	5.4 × 10^−6^	0.80
Nb	14	3 × 10^6^	5.1 × 10^−7^	0.99	1 × 10^3^	5.4 × 10^−6^	0.87

## Data Availability

The data presented in this study are available on request from the corresponding author.

## References

[1] Young L. (1961). Anodic Oxide Films.

[2] Lohrengel M.M. (1993). Thin Anodic Oxide Layers on Aluminium and Other Valve Metals: High Field Regime. Mater. Sci. Eng. R Rep..

[3] di Franco F., Zaffora A., Santamaria M., di Quarto F., Wandelt K. (2018). Anodization and Anodic Oxides. Encyclopedia of Interfacial Chemistry: Surface Science and Electrochemistry.

[4] Robertson J., Wallace R.M. (2015). High-K Materials and Metal Gates for CMOS Applications. Mater. Sci. Eng. R Rep..

[5] Zaffora A., di Franco F., Santamaria M., Habazaki H., di Quarto F. (2015). The Influence of Composition on Band Gap and Dielectric Constant of Anodic Al-Ta Mixed Oxides. Electrochim. Acta.

[6] Franke E., Trimble C.L., DeVries M.J., Woollam J.A., Schubert M., Frost F. (2000). Dielectric Function of Amorphous Tantalum Oxide from the Far Infrared to the Deep Ultraviolet Spectral Region Measured by Spectroscopic Ellipsometry. J. Appl. Phys..

[7] Guo Y., Robertson J. (2014). Oxygen Vacancy Defects in Ta_2_O_5_ Showing Long-Range Atomic Re-Arrangements. Appl. Phys. Lett..

[8] French R.H. (1990). Electronic Band Structure of Al_2_O_3_, with Comparison to Alon and AIN. J. Am. Ceram. Soc..

[9] Gaskins J.T., Hopkins P.E., Merrill D.R., Bauers S.R., Hadland E., Johnson D.C., Koh D., Yum J.H., Banerjee S., Nordell B.J. (2017). Review—Investigation and Review of the Thermal, Mechanical, Electrical, Optical, and Structural Properties of Atomic Layer Deposited High- k Dielectrics: Beryllium Oxide, Aluminum Oxide, Hafnium Oxide, and Aluminum Nitride. ECS J. Solid State Sci. Technol..

[10] Scaduto G., Santamaria M., Bocchetta P., di Quarto F. (2014). The Effect of Hydration Layers on the Anodic Growth and on the Dielectric Properties of Al_2_O_3_ for Electrolytic Capacitors. Thin Solid Films.

[11] di Franco F., Santamaria M., di Quarto F., la Mantia F., de Sá A.I., Rangel C.M. (2013). Dielectric Properties of Al-Nb Amorphous Mixed Oxides. ECS J. Solid State Sci. Technol..

[12] di Franco F., Zampardi G., Santamaria M., di Quarto F., Habazaki H. (2012). Characterization of the Solid State Properties of Anodic Oxides on Magnetron Sputtered Ta, Nb and Ta-Nb Alloys. J. Electrochem. Soc..

[13] Mardare A.I., Ludwig A., Savan A., Hassel A.W. (2014). Electrochemistry on Binary Valve Metal Combinatorial Libraries: Niobium-Tantalum Thin Films. Electrochim. Acta.

[14] Komiyama S., Tsuji E., Aoki Y., Habazaki H., Santamaria M., di Quarto F., Skeldon P., Thompson G.E. (2012). Growth and Field Crystallization of Anodic Films on Ta–Nb Alloys. J. Solid State Electrochem..

[15] Zaffora A., di Quarto F., Habazaki H., Valov I., Santamaria M. (2019). Electrochemically Prepared Oxides for Resistive Switching Memories. Faraday Discuss..

[16] Vorobjova A.I., Tishkevich D.I., Outkina E.A., Shimanovich D.L., Razanau I.U., Zubar T.I., Bondaruk A.A., Zheleznova E.K., Dong M., Aloraini D.A. (2022). A Study of Ta2O5 Nanopillars with Ni Tips Prepared by Porous Anodic Alumina Through-Mask Anodization. Nanomaterials.

[17] Tishkevich D.I., Vorobjova A.I., Vinnik D.A. (2020). Formation and Corrosion Behavior of Nickel/Alumina Nanocomposites. Solid State Phenom..

[18] Banerjee P., Perez I., Henn-Lecordier L., Lee S.B., Rubloff G.W. (2009). Nanotubular Metal–Insulator–Metal Capacitor Arrays for Energy Storage. Nat. Nanotechnol..

[19] Kim Y.-H., Uosaki K. (2013). Preparation of Tantalum Anodic Oxide Film in Citric Acid Solution—Evidence and Effects of Citrate Anion Incorporation. J. Electrochem. Sci. Technol..

[20] Ono S., Kuramochi K., Asoh H. (2009). Effects of Electrolyte PH and Temperature on Dielectric Properties of Anodic Oxide Films Formed on Niobium. Corros Sci..

[21] di Franco F., Zaffora A., Santamaria M. (2018). Band Gap Narrowing and Dielectric Constant Enhancement of (Nb_x_Ta_(1-x)_)_2_O_5_ by Electrochemical Nitrogen Doping. Electrochim. Acta.

[22] Zaffora A., di Franco F., di Quarto F., Santamaria M. (2020). Optimization of Anodizing Process of Tantalum for Ta2O5-Based Capacitors. J. Solid State Electrochem..

[23] Pourbaix M. (1966). Atlas of Electrochemical Equilibria in Aqueous Solutions.

[24] Zaffora A., di Franco F., di Quarto F., Macaluso R., Mosca M., Habazaki H., Santamaria M. (2017). The Effect of Nb Incorporation on the Electronic Properties of Anodic HfO_2_. ECS J. Solid State Sci. Technol..

[25] di Quarto F., Gentile C., Piazza S., Sunseri C. (1993). A Photoelectrochemical Study on Anodic Tantalum Oxide Films. Corros Sci..

[26] Mott N.F., Davis E.A. (1979). Electronic Processes in Non-Crystalline Materials.

[27] di Franco F., Santamaria M., di Quarto F., Tsuji E., Habazaki H. (2012). The Influence of Nitrogen Incorporation on the Optical Properties of Anodic Ta_2_O_5_. Electrochim. Acta.

[28] Zaffora A., Santamaria M., di Franco F., Habazaki H., di Quarto F. (2016). Photoelectrochemical Evidence of Nitrogen Incorporation during Anodizing Sputtering-Deposited Al-Ta Alloys. Phys. Chem. Chem. Phys..

[29] Zaffora A., Santamaria M., di Franco F., Habazaki H., di Quarto F. (2016). Photoelectrochemical Evidence of Inhomogeneous Composition at Nm Length Scale of Anodic Films on Valve Metals Alloys. Electrochim. Acta.

[30] Shimizu K., Habazaki H., Skeldon P., Thompson G.E., Wood G.C. (2001). Migration of Oxalate Ions in Anodic Alumina. Electrochim. Acta.

[31] Nishio K., Sasaki R. (2019). Anodization of Al in Neutral Oxalate Solution to Form Unusually Fine Nanoporous Film. Chem. Lett..

[32] Brudzisz A.M., Giziński D., Stępniowski W.J. (2021). Incorporation of Ions into Nanostructured Anodic Oxides—Mechanism and Functionalities. Molecules.

[33] Shimizu K., Habazaki H., Skeldon P., Thompson G.E. (2003). Radiofrequency GDOES: A Powerful Technique for Depth Profiling Analysis of Thin Films. Surf. Interface Anal..

[34] Habazaki H., Teraoka M., Aoki Y., Skeldon P., Thompson G.E. (2010). Formation of Porous Anodic Titanium Oxide Films in Hot Phosphate/Glycerol Electrolyte. Electrochim. Acta.

[35] Habazaki H., Fushimi K., Shimizu K., Skeldon P., Thompson G.E. (2007). Fast Migration of Fluoride Ions in Growing Anodic Titanium Oxide. Electrochem. Commun..

[36] Habazaki H., Uozumi M., Konno H., Shimizu K., Skeldon P., Thompson G.E. (2003). Crystallization of Anodic Titania on Titanium and Its Alloys. Corros Sci..

[37] Lu Q., Skeldon P., Thompson G.E., Masheder D., Habazaki H., Shimizu K. (2004). Transport Numbers of Metal and Oxygen Species in Anodic Tantala. Corros Sci..

